# Inferring connectivity of an oscillatory network via the phase dynamics reconstruction

**DOI:** 10.3389/fnetp.2023.1298228

**Published:** 2023-11-23

**Authors:** Michael Rosenblum, Arkady Pikovsky

**Affiliations:** Institute of Physics and Astronomy, University of Potsdam, Potsdam, Germany

**Keywords:** oscillations, network, connectivity, data analysis, phase reduction

## Abstract

We review an approach for reconstructing oscillatory networks’ undirected and directed connectivity from data. The technique relies on inferring the phase dynamics model. The central assumption is that we observe the outputs of all network nodes. We distinguish between two cases. In the first one, the observed signals represent smooth oscillations, while in the second one, the data are pulse-like and can be viewed as point processes. For the first case, we discuss estimating the true phase from a scalar signal, exploiting the protophase-to-phase transformation. With the phases at hand, pairwise and triplet synchronization indices can characterize the undirected connectivity. Next, we demonstrate how to infer the general form of the coupling functions for two or three oscillators and how to use these functions to quantify the directional links. We proceed with a different treatment of networks with more than three nodes. We discuss the difference between the structural and effective phase connectivity that emerges due to high-order terms in the coupling functions. For the second case of point-process data, we use the instants of spikes to infer the phase dynamics model in the Winfree form directly. This way, we obtain the network’s coupling matrix in the first approximation in the coupling strength.

## 1 Introduction

### 1.1 The connectivity problem

Inferring network connectivity from observation represents one of the most challenging data analysis problems. This task finds practical applications in many fields and particularly in life sciences. For example, determining brain area connectivity is essential for studying normal and pathological brain function. This brief review summarizes one of the existing approaches to the connectivity problem. This approach is not general: it applies to the case of oscillatory networks when each node represents an active, self-sustained oscillator. However, as we argue below, the approach is natural for this case, and the results admit a clear interpretation. The technique relies on the phase dynamics reconstruction from observations; it assumes that the outputs of all network nodes are available.

Before proceeding with the approach’s description and discussion, we briefly formulate how we understand the connectivity. We start by formulating an ensemble of *N* coupled dynamical systems, the couplings are not necesseraly pairwise, but can include many-body interactions. Suppose the dynamics of the *k*th node are described by ${\dot{x}}_{k}=F_{k}\left(x_{k}\right)+\varepsilon H_{k}\left(x_{k},x_{1},\ldots ,x_{N}\right)$ , where the vector **x**_*k*_ consists of the state variables at the node *k* (below we focus on the case of weak coupling, thus we introduce a parameter *ɛ* having the dimension of frequency). Notice that even a more generic setup could be considered, where couplings themselves are described by some differential equations. Vector functions **F**_*k*_ and **H**_*k*_ determine the autonomous and the interaction dynamics of the node *k*. Since the numbering of oscillators is arbitrary, for the sake of brevity it is convenient to write, without loss of generality, the dynamics equation for the first unit, i.e., to set *k* = 1 and omit one index. Thus, we write

$$\dot{x}=F\left(x\right)+\varepsilon H\left(x,x_{2},\ldots ,x_{N}\right).$$ (1)

This equation and its phase approximation version written below help to quantify all incoming links 1 ← *j*, *j* = 2, … , *N*. If the coupling term **H** depends explicitly^1^ on **x**_*j*_, we say that there exists a structural link *j* → 1. In other words, a structural link means some physical connection, e.g., resistive coupling of electronic circuits, synaptic or gap-junction neuronal coupling, connection of brain areas via fibers, etc. Such connections are represented by an explicit dependence in the dynamical equations, which is generally directional (i.e., existing connection *j* → 1 does not imply the link 1 → *j* and *vice versa*; if both links exist, their strengths generally differ). Naturally, node 1 can have many incoming links. In this case, the function **H** depends on many arguments. Below, we mainly concentrate on the typical case of pair-wisely coupled networks when

$$H=\underset{j\neq 1}{\sum }H_{j}\left(x,x_{j}\right),$$ (2)

but in Section 2.3.4, also many-body (triplet) couplings will be discussed shortly.

Even if the nodes *k*, *j* are not structurally connected, they may exhibit correlated dynamics due to a common drive or indirect coupling via a third node, etc. Different measures partially discussed below quantify the degree of correlation or, in other words, the functional connectivity. To illustrate the difference between structural and functional connectivities, consider a simple motif of three oscillators, where the second unit drives the other two, 1 ← 2 → 3. Obviously, units 1 and 3 are not structurally linked but may be correlated, i.e., functionally connected.

Most important for us is the notion of effective phase connectivity because it is precisely what the approach discussed below yields. A characterization of oscillations with their phases (to be discussed in more details in Section 1.2 below) replaces dynamical variables at each node **x**_*k*_ with the phases *φ*_*k*_. Generally, the phase dynamics equation of the first node reads

$$\dot{\varphi }=\omega +\varepsilon h\left(\varphi ,\varphi_{2},\ldots ,\varphi_{N}\right),$$ (3)

where *ω* is the natural frequency, and *h* is the (phase) coupling function. If *h* depends on *φ*_*j*_, we say that there is an effective phase connection *j* → 1. We discuss the relation between functions **H** and *h* and the relation between structural and effective phase connectivities in the next section after recalling the basic results of the phase reduction theory. We emphasize that in the context of the connectivity problem, the word “effective” sometimes means the data-based estimation of the true connectivity. We use the term to describe the connectivity as the true phase dynamics give it; as we argue below, it generally differs from structural connectivity.

### 1.2 Basic facts about phase reduction

As already emphasized, the reviewed approach applies to networks of self-sustained units. Although natural oscillators are inevitably noisy or weakly chaotic, many analysis techniques rely on the phase reduction theory, which is mostly easily formulated for coupled limit-cycle oscillators (and then extended under some approximations to other cases). In this Section, we briefly present the main results of this theory. Thus, here we assume that isolated systems possess stable limit cycles and, hence, exhibit periodic oscillations. It is well-known that for such systems the phase variable can be introduced obeying

$${\dot{\varphi }}_{k}=\omega_{k}.$$ (4)

It is important that the phase is defined both for the limit cycle and its basin of attraction. The description of the dynamics in terms of the phases means an immediate reduction of the problem dimensionality: each limit-cycle oscillator, two- or multidimensional, is now quantified by only one variable *φ*_*k*_. The dynamics of interacting units obeys Eq. 3 and the phase space of the system is the *N*-dimensional torus, unless the coupling becomes too strong and destroys the torus (Afraimovich and Shilnikov, 1983). We underline that derivation of functions *h* from given **H** remains an unsolved problem in the general case. The well-known theory provides a recipe for the derivation in the first-order approximation in the interaction strength, and even in this case the analytical solution requires knowledge of phase sensitivity functions [see detailed discussion by Nakao (2016)]. However, for network reconstruction from data, this derivation is not needed. We only need the general properties of functions *h*.

Let us assume that the coupling strength *ɛ* is small compared to the frequency *ɛ* ≪ *ω*, and express the function *h* in powers of *ɛ*. Eq. 3 becomes

$$\dot{\varphi }=\omega +\varepsilon Q_{1}\left(\varphi ,\varphi_{2},\ldots ,\varphi_{N}\right)+\varepsilon^{2}Q_{2}\left(\varphi ,\varphi_{2},\ldots ,\varphi_{N}\right)+\cdots \;.$$ (5)

In the limit of weak coupling, one keeps only the first-order term *Q*_1_, and the general theory says, that for the pair-wise coupling, see Eq. 2, this term reads

$$Q_{1}\left(\varphi ,\varphi_{2},\ldots ,\varphi_{N}\right)=\underset{j>1}{\sum }q_{j}\left(\varphi ,\varphi_{j}\right).$$ (6)

It means that in the weak-coupling limit, the pair-wise coupling on the level of full equations results in the pair-wise coupling in the phase model. This property is not preserved if the high-order terms, i.e., terms ∼*ɛ* ^2^, ∼*ɛ* ^3^, … are not negligible. So, the analysis of the analytically solvable case of three oscillators coupled pair-wisely in a chain (i.e., only structural couplings 1 ↔ 2 and 2 ↔ 3 are present) yields the second-order term *Q*_2_(*φ*, *φ*_2_, *φ*_3_) in the equation for the first unit dependent on all three phases (Gengel et al., 2021), though the first and third oscillators are not structurally linked: they interact only through the second oscillator. This theoretical result confirms earlier numerical observations (Kralemann et al., 2011; Kralemann et al., 2014) demonstrating the difference between the structural and effective phase connectivity. We expect that third-order terms *Q*_3_ provide dependence on the phase of the *l*th unit interacting with the first oscillator indirectly through two mediators, e.g., in the following configuration, 1 ↔ *n* ↔ *m* ↔ *l*, and so on.

A remark is in order. Consider for a moment two oscillators. The first-order phase approximation yields a general function of two phases, *Q*_1_ = *Q*_1_(*φ*_1_, *φ*_2_). If the coupling is weak *ɛ* ≪ *ω*, then one typically performs another approximation step, by averaging the phase equation over the oscillation period 2*π*/*ω* and obtaining the averaged coupling function (describing slow variations of the phases) that depends on the slow phase difference only, ${\tilde{Q}}_{1}={\tilde{Q}}_{1}\left(\varphi_{1}-\varphi_{2}\right)$ (the Kuramoto-Daido form), see, e.g., Pikovsky et al. (2001). This step essentially facilitates the theoretical treatment of the phase dynamics, but using the Kuramoto-Daido form instead of the general dependence may be an unnecessary simplification for the connectivity inference from data. Another useful representation of the phase dynamics is the Winfree form. If the term **H**_2_ can be written as **H**_2_(**x**_1_, **x**_2_) = **s** *H*_2_(**x**_1_, **x**_2_) = **s** *h*_2_(*φ*_1_, *φ*_2_), where **s** is a constant unit vector and we substituted the variables **x**_1,2_ on the limit cycles as functions of *φ*_1,2_, then one obtains

$$Q_{1}\left(\varphi_{1},\varphi_{2}\right)=Z\left(\varphi_{1}\right)h_{2}\left(\varphi_{1},\varphi_{2}\right),$$ (7)

where *Z*(*φ*_1_) is the phase sensitivity function for a perturbation in direction **s**, or phase response curve (PRC). In many cases one assumes that the driving term does not contain the driven coordinates, i.e., *h*_2_(*φ*_2_) contains the driving phase only. In case of more than two units, the sensitivity functions generally differ for different inputs.

All real-world oscillators are noisy, and in the simplest case of a state-independent noise we shall add a random term to the right-hand side of Eqs 1–5. However, the main properties of the phase dynamics remain, also for the case of weakly chaotic oscillators.

In summary, the main idea of the approach reviewed in this paper is to estimate phases from observed time-series data and reconstruct Eq. 3 for each network node. These equations provide effective phase connectivity that is close but not identical to the structural one. Two factors render efficient reconstruction: 1) phase representation is low-dimensional (there is only one equation per unit), and 2) the function *h* on the right-hand side has a relatively simple form (it can be represented as a multiple Fourier series). This inference is possible if the data are suitable for phase estimation; we discuss this case in the next section. The situation differs if the data look like a sequence of spikes. We address this problem in Section 3.

## 2 Case I: smooth oscillatory dynamics

### 2.1 From time series to phases

#### 2.1.1 From time series to a protophase

The first step in the phase dynamics analysis is the phase inference from the observed signals. Generally, observables are functions of the state variables **x**. For periodic dynamics (i.e., without coupling) these observables are periodic functions of time, but under coupling, these observables are nearly periodic signals with amplitude and phase modulations. An extraction of the phase is simple, if at least two scalar observables $y_{k}^{\left(1\right)}\left(t\right),y_{k}^{\left(2\right)}\left(t\right)$ for a system *k* are available. In this case, a two-dimensional plot of a trajectory on the plane (*y* ^(1)^, *y* ^(2)^) is a nearly closed curve, and one can characterize the phase by a coordinate along the curve. In the simplest case when the curve is close to a circle, one can take $\theta_{k}=\text{arg}\left[y_{k}^{\left(1\right)}-\left\langle y_{k}^{\left(1\right)}\right\rangle +i\left(y_{k}^{\left(2\right)}-\left\langle y_{k}^{\left(2\right)}\right\rangle \right)\right]$ as such a coordinate. If the curve is more complex, e.g., it has several loops, one can define this coordinate as a normalized length along the curve,

$$\theta =2\pi \frac{L\left(t\right)-L\left(t_{i}\right)}{L\left(t_{i+1}\right)-L\left(t_{i}\right)}$$

(here *t*_*i*_ are time instants at which the *i*th revolution completes; each revolution corresponds to one oscillation). This approach ensures that the obtained coordinate *θ* grows monotonically with time. A complex waveform appears, e.g., in the analysis of an electrocardiogram, where one heartbeat cycle consists of several “waves.” For an advanced algorithm developed for the ECG analysis, see Kralemann et al. (2013a).

The problem is less trivial if only one scalar observable *y*_*k*_(*t*) is available. To perform the embedding, one needs to construct from it the second observable. The method of choice in the literature is obtaining the second time series by virtue of the Hilbert Transform (King, 2009; Feldman, 2011): ${\tilde{y}}_{k}\left(t\right)=\hat{H}\left[y_{k}\left(t\right)\right]$ . The variable *θ* based on the embedding $\left(y_{k}\left(t\right),{\tilde{y}}_{k}\left(t\right)\right)$ is often called “Hilbert phase” (quite often it is additionally assumed “by default” that *θ* is an argument of a complex observable $y_{k}\left(t\right)+i\hat{H}\left[y_{k}\left(t\right)\right]$ ). This method works reasonably well for a nearly harmonic signal with a weak and slow modulation of the phase and small amplitude modulation. If the phase modulation is not slow and small, the HT method is inaccurate but still used in many applications because of its simplicity. Recently, an improvement of the HT method has been suggested (Gengel and Pikovsky, 2019; Gengel and Pikovsky, 2021; Gengel and Pikovsky, 2022). The procedure implies an iterative approach: one takes the variable *θ* ^(1)^ based on the embedding $\left(y_{k}\left(t\right),\hat{H}\left[y_{k}\left(t\right)\right]\right)$ as a first approximation (thus the superscript 1), and uses it as a new time variable to perform a new embedding $\left(y_{k}\left(\theta^{\left(1\right)}\right),\hat{H}\left[y_{k}\left(\theta^{\left(1\right)}\right)\right]\right)$ , where now in the computation of HT one considers the signal as a function of *θ* ^(1)^ instead of a function of time. This step begets a new, improved coordinate *θ* ^(2)^, etc. Gengel and Pikovsky (2019), Gengel and Pikovsky (2021), and Gengel and Pikovsky (2022) demonstrated that for a signal with a pure phase modulation, this iterative procedure converges, allowing for a perfect inference of a phase. However, if amplitude modulation is also present, the inference is inaccurate. Developing a reliable method for phase and amplitude demodulation of a signal remains a challenging problem; see also Matsuki et al. (2023).

The variable *θ* discussed above is monotonic in time and grows by 2*π* at each oscillation, but it is not the phase because the genuine phase of an oscillator has a special property—it grows, according to Eq. 4, uniformly in time, while the variable *θ* obeys

$$\dot{\theta }=\omega +f\left(\theta \right),$$ (8)

where the function *f*(*θ*) depends on the form and selection of the observables *y* and on the method of its extraction. To emphasize this difference, variable *θ* is called *protophase*.

#### 2.1.2 From a protophase to the phase

The transformation from a monotonic protophase to the true phase is a necessary and straightforward step in the phase reduction theory^2^. Writing $\frac{d\varphi }{dt}=\omega =\frac{d\varphi }{d\theta }\frac{d\theta }{dt}$ , one obtains the desired transformation

$$\frac{d\varphi }{d\theta }=\omega {\left[\frac{d\theta }{dt}\right]}^{-1}=\sigma \left(\theta \right),\;\text{or}\;\varphi =\int_{0}^{\theta }\sigma \left(\theta^{\prime }\right)d\theta^{\prime }.$$ (9)

Finding the transformation function *σ*(*θ*) in data analysis is non-trivial: we must estimate it from a noisy trajectory. Hence, we must compute an average of d*t*/d*θ* over the trajectory. Kralemann et al. (2007) and Kralemann et al. (2008) demonstrate an efficient algorithm that avoids numerical differentiation. Let the discretely sampled protophases be ${\hat{\theta }}_{j}$ , *j* = 1, … , *N*_*p*_, where *N*_*p*_ is the number of points. Then the transformation reads:

$$\varphi =\theta +2\overset{M}{\underset{n=1}{\sum }}\text{Im}\left[S_{n}\left(e^{in\theta }-1\right)/n\right],\;\text{where}\;S_{n}=N_{p}^{-1}\overset{N_{p}}{\underset{j=1}{\sum }}e^{in{\hat{\theta }}_{j}}.$$ (10)

Here *M* should not be chosen too high to avoid overfitting. Numerical tests by Kralemann et al. (2008) demonstrate that the protophase-to-phase transformation (Eq. 10) provides a nearly homogeneously growing phase. It removes the deviations from *ωt* with the characteristic time 2*π*/*ω*, but preserves low-frequency features like phase diffusion. It is important that the transformation (Eq. 10) is invertible and therefore not a “filtering” or “smoothing.”

### 2.2 Undirected connectivity

The main qualitative effect of weak interaction between self-oscillatory systems is a possibility of their synchronization manifested by frequency and phase locking. So, for two periodic oscillators, which, being uncoupled, have close but different frequencies, synchronization means that the frequencies become precisely equal if the interaction is switched on and its strength exceeds some threshold. Equality of frequencies means boundness of the phase difference *ψ* = *φ*_1_ − *φ*_2_, i.e., |*ψ*| < const. Generally, if the frequencies of uncoupled systems fulfill *nω*_1_ ≈ *mω*_2_, where *n*, *m* are some positive integers, the interaction may result in synchronization, which can be defined according to frequency and phase locking conditions, *n*Ω_1_ = *m*Ω_2_ and |*ψ*_*n*,*m*_| = |*nφ*_1_ − *mφ*_2_| < const, respectively; here, Ω_1,2_ denote frequencies of coupled systems (observed frequencies). In a noisy environment, *ψ*_*n*,*m*_ remains constant for long time intervals, but can exhibit relatively rapid ± 2*π* jumps, called phase slips. Then, the locking condition |*ψ*| < const does not hold, but the distribution of *ψ*_*n*,*m*_ mod 2*π* remains non-uniform. Note that phase slips also appear in a noise-free case when the oscillators are slightly outside the border of synchrony. A measure of the non-uniformity of the distribution of the phase differences quantifies thus phase coherence. In other words, it quantifies how far the system is from the asynchronous case of two uncoupled oscillators where the distribution of *ψ*_*n*,*m*_ mod 2*π* is uniform.

The corresponding measure can be the entropy of the distribution or the amplitude of its first Fourier mode. The latter is known as the phase locking value or synchronization index and is most popular because it has no parameters (Rodriguez et al., 1999; Mormann et al., 2000; Rosenblum et al., 2001). For the general case of *n*: *m* locking, where *n*, *m* are some positive integers, this measure is

$$\gamma_{n,m}=|\left\langle \exp\left[i\left(n\varphi_{1}-m\varphi_{2}\right)\right]\right\rangle |,$$ (11)

where ⟨⋅⟩ means averaging over time. Obviously, 0 ≤ *γ*_*n*,*m*_ ≤ 1; this index quantifies the constancy of the phase difference. Since the ratio between the frequencies of the uncoupled systems is unknown, though it can be roughly estimated, one tries different combinations of *n*, *m* and picks the values which maximize *γ*_*n*,*m*_.

For a network, computing the index *γ*_*n*,*m*_, we can assign its value to the link between these nodes. This procedure provides an undirected network of functionally connected units but does not yield reliable information about the underlying physical connections. To illustrate this issue, let us consider a motif of three units coupled in the following configuration: 1 ↔ 2 ↔ 3. It is known that the 1st and 3rd oscillators can synchronize, while the central unit 2 remains asynchronous. This state is denoted as remote synchrony, see Bergner et al. (2012); its appearance can be explained by the second-order phase reduction that yields the coupling term ∼*ɛ* ^2^ dependent on *φ*_1_ − *φ*_3_ (Kumar and Rosenblum, 2021). For this state, quantifying functional interrelations through the index *γ* will yield a strong link 1 ↔ 3 and no links for the pairs (1, 2) and (2, 3).

**FIGURE 1:**
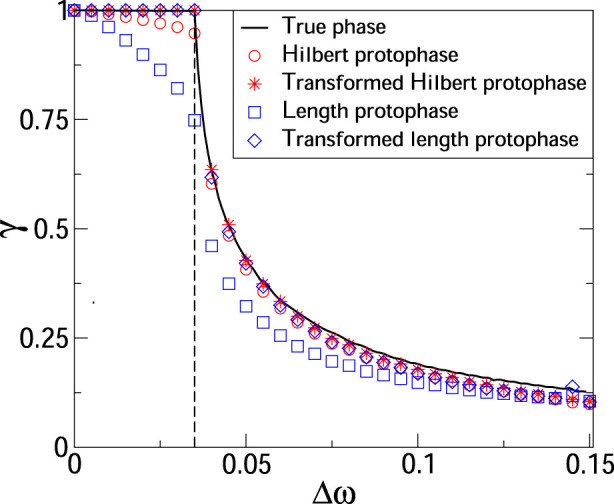
Synchronization index as a function of frequency mismatch in a system of two coupled van der Pol oscillators, see Eq. 12. The solid black line shows the index computed from the true phases; this computation requires knowledge of the systems equations and serves as a benchmark. The vertical dashed line marks the border of the synchronization domain. We see that computing *γ* from protophases 1) yields the protophase-dependent results, and 2) the index value can be essentially smaller than one for synchronous states. Next, we see that the protophase-to-phase transformation provides an essential improvement.

With Figure 1 we illustrate the importance of the protophase-to-phase transformation for computation of *γ*. See Kralemann et al. (2008) for analytical examples demonstrating that calculating the index from protophases can yield either an under- or overestimated value. Here, we generate the signals by simulating the dynamics of two coupled van der Pol oscillators:

$${\ddot{x}}_{1,2}-3\left(1-x_{1,2}^{2}\right){\dot{x}}_{1,2}+{\left(1\pm \Delta \omega \right)}^{2}x_{1,2}=0.1\left({\dot{x}}_{2,1}-{\dot{x}}_{1,2}\right).$$ (12)

Taking *x*_1,2_ as observables, we compute the Hilbert protophases $\theta_{1,2}^{\left(H\right)}$ and length protophases $\theta_{1,2}^{\left(L\right)}$ . Then we compute *γ*_1,1_ from protophases and transformed protophases. For the benchmark, we take the index calculated from true phases^3^. The results demonstrate that the index *γ* depends on the chosen protophase, while the protophase-to-phase transformation eliminates this ambiguity and crucially improves the estimate. Finally, we mention that the index is large outside of the synchronization domain, which means that it is not a measure of synchronization but of the interaction strength reflected in phase correl ations.

The definition of the synchronization index can be easily extended to quantify triplet interactions. Consider triplet locking defined via the condition

$$|n\varphi_{1}+m\varphi_{2}+l\varphi_{3}|<\text{const},\;n\omega_{1}+m\omega_{2}+l\omega_{3}=0,$$ (13)

where integers *n*, *m*, *l* can be both positive and negative, while the conditions of the pairwise locking are not satisfied for any pair of units (Kralemann et al., 2013b). The corresponding index is

$$\gamma_{n,m,l}=|\left\langle \exp\left[i\left(n\varphi_{1}+m\varphi_{2}+l\varphi_{3}\right)\right]\right\rangle |.$$ (14)

We emphasize that many-body interaction when coupling terms depend on more than two phases naturally appears in oscillatory networks, see Pikovsky and Rosenblum (2022) for a review and Nijholt et al. (2022) for an experimental illustration.

A natural question is: what is the advantage of index *γ*_*n*,*m*_ over the usual correlation directly computed from the time series without phase estimation? The reason is twofold. First, the synchronization index distinguishes the locking states (or, more precisely, states close to locking) of different orders. Second, if the oscillators are noisy or weakly chaotic, the amplitudes may strongly fluctuate while the phases can lock. Thus, a measure exploiting phases is more sensitive to interaction.

### 2.3 Directed connectivity

Determination of the true structural connectivity of a network requires knowledge of the state space Eq. 1. One can compute the norm of the corresponding coupling function **H** as a measure of the connection’s strength. So the effect of the *j*th node on the first one is quantified by ‖**H**(**x**, **x**_*j*_)‖. However, such quantification is not complete since the effect of the forcing depends not only on the force’s strength but also on the system’s sensitivity to this influence. Anyway, reconstruction of Eq. 1 from scalar data is hardly feasible. In contradistinction, inference of the phase dynamics Eq. 3 is possible; some limitations are discussed below. In the rest of this section, we treat separately the cases of two, three, and more than three units.

#### 2.3.1 Two oscillators

Suppose we observe two oscillators and register two observables *y*_1,2_. Suppose also that these observables are suitable for phase estimation. Then, we perform a two-step procedure for each observable: first, we compute protophases exploiting an appropriate technique and then execute the protophase-to-phase transformation. The next step is to estimate phase derivatives (the instantaneous frequencies). To this end, we first unwrap each phase to make it a monotonically growing function of time and then apply a Savitzky–Golay filter, i.e., for each time point, perform a local polynomial fit in a running window, and obtain the derivative at this point from the fitted curve. Local fitting is a smoothing filter, making this approach especially useful for noisy data. Having phases and their derivatives, we find the right-hand sides of coupled equations

$${\dot{\varphi }}_{1,2}=\omega_{1,2}+Q^{\left(1,2\right)}\left(\varphi_{1,2},\varphi_{2,1}\right)$$

(here, it is convenient to write the index of the first system explicitly)^4^. There are at least two ways to achieve this. The first one is to use the fact that the coupling function is 2*π*-periodic in both arguments, represent it by a double Fourier series, and find the coefficient of this series by the least mean squares fit. The second one is to use the kernel density estimation to obtain the desired function on a rectangular grid. Note that practically we compute the r.h.s. of phase equations and then represent it as a sum of the constant non-zero term (frequency *ω*) and zero-mean function^5^.

Now, we introduce an index *d*_1→2_ quantifying the directionality of coupling (Rosenblum and Pikovsky, 2001). It is defined so that *d*_1→2_ = 1 for unidirectional coupling from the first to the second unit, *d*_1→2_ = −1 for the opposite case, and −1 ≤ *d*_1→2_ ≤ 1 for bidirectional coupling. The directionality index is

$$d_{1\to 2}=\frac{c_{2}-c_{1}}{c_{1}+c_{2}},$$

where *c*_1,2_ = ‖*Q* ^(1,2)^‖/*ω*_1,2_ and $\|Q^{\left(1,2\right)}{\|}^{2}=\sum |F_{n,m}^{\left(1,2\right)}{|}^{2}$ , where $F_{n,m}^{\left(1,2\right)}$ are the Fourier coefficients of the corresponding zero-mean function *Q* ^(1,2)^. Coefficients *c*_1,2_ provide a relative dimensionless measure of the external action on a unit. For effects of noise and data length on the efficiency of this technique, see Smirnov and Bezruchko (2003). Comparison of this approach with the partial directed coherence can be found in Smirnov et al. (2007).

We conclude the discussion of the two-units case with two remarks. 1) Inference of a function of two variables *φ*_1_, *φ*_2_ requires that the given points scatter over the square domain (toroidal surface) 0 ≤ *φ*_1,2_ < 2*π*. In other words, the systems shall not synchronize. In the opposite case, the trajectory is just a curve on the surface of the torus, and the fitting procedure fails. 2) A widely used approach is reconstructing the coupling function as a function of the phase difference, i.e., in the Kuramoto-Daido form. Such inference requires less numerical effort than recovering the general function of two phases, but the price is that one has to choose the *n*: *m* ratio beforehand.

#### 2.3.2 Three oscillators

For a motif of three oscillators, we first calculate three protophases, then perform three times the transformation to phases, and infer the coupling functions exploiting the least mean squares fit^6^. To be exact, by fitting, one finds the coefficients of the Fourier representation, e.g., for the first oscillator:

$${\dot{\varphi }}_{1}=\underset{l_{1},l_{2},l_{3}}{\sum }F_{l_{1},l_{2},l_{3}}\exp\left[i\left(l_{1}\varphi_{1}+l_{2}\varphi_{2}+l_{3}\varphi_{3}\right)\right].$$

Then we set $\omega =F_{0,0,0}$ and assign the sum of all terms except for the constant one to *Q* ^(1)^. To quantify a link, e.g., a pairwise action from 2 → 1, we compute a partial norm

$$P_{1\leftarrow 2}^{2}=\underset{l_{1},l_{2}\neq 0}{\sum }|F_{l_{1},l_{2},0}{|}^{2}.$$ (15)

Quantification of the joint action of units 2 and 3 on the first one is given by

$$T_{1\leftarrow 2,3}^{2}=\underset{l_{1},l_{2}\neq 0,l_{3}\neq 0}{\sum }|F_{l_{1},l_{2},l_{3}}{|}^{2}.$$ (16)

We emphasize that one can perform a pairwise analysis, e.g., reconstructing the coupling function *Q* ^(1)^ from phases *φ*_1_, *φ*_2_ as described in the previous section. In this way, one ignores the third oscillator. The norm of the reconstructed function ‖*Q* ^(1)^‖ generally differs from $P_{1\leftarrow 2}$ ; indeed, the former one captures both direct and indirect causal effect from 2 to 1, while the latter is less sensitive to indirect effects.

#### 2.3.3 More than three oscillators

**FIGURE 2:**
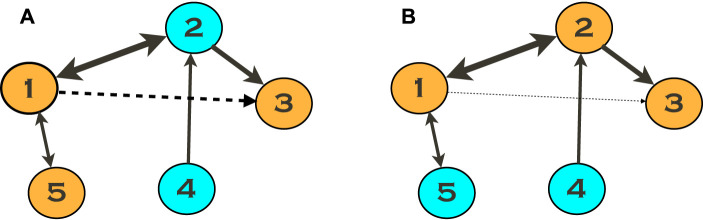
Triplet analysis of networks with *N* > 3 oscillators. Suppose we quantify the link 1 → 3 absent in the given configuration. However, if we estimate the link strength using Eq. 17 exploiting the triplet (3, 1, 5), shown by the orange color in **(A)**, we obtain a non-zero value, indicated by a dashed line. The explanation is that units 1 and 3 are connected through unit 2, and this connection is not captured by the triplet used. We obtain a much better estimation using the triplet (3, 1, 2) **(B)**. Notice that the resulting measure is always positive. Therefore, taking minimum over all triplets, see Eq. 18, yields the best estimation.

An extension to the case of *N* > 3 units seems straightforward. However, by reconstructing a high-dimensional coupling function, we face the curse of dimensionality. For a successful inference, we must fill the *N*-dimensional hypercube, which requires large data sets. A possible solution is to use triplet analysis (Kralemann et al., 2014). Suppose we want to quantify a link from unit *k* to unit *j*. To this goal, we reconstruct the equation

$${\dot{\varphi }}_{j}=\omega_{j}+Q^{\left(j,k,m\right)}\left(\varphi_{j},\varphi_{k},\varphi_{m}\right)$$

for all *m* ≠ *j*, *k*, i.e., for all possible triplets. Then, from each triplet we obtain partial norm

$${\tilde{T}}_{j\leftarrow k}^{2}\left(m\right)=\underset{l_{j},l_{k}\neq 0}{\sum }{\left|F_{l_{j},l_{k},0}^{\left(j\right)}\right|}^{2}$$ (17)

and take

$$T_{j\leftarrow k}^{2}=\underset{m}{\min}{\tilde{T}}_{j\leftarrow k}^{2}\left(m\right)$$ (18)

as the final triplet-based measure of the link strength. Figure 2 illustrates the idea.

Numerical experiments (Kralemann et al., 2014) with small networks (*N* = 5 and *N* = 9) demonstrate that the triplet analysis provides a very good separation between existing and non-existing connections. Moreover, the results confirm that most of the non-existing links revealed by the technique are not artifacts but reflect causal information flow mediated by indirect driving. This driving is due to terms emerging in the high-order phase reduction.

The technique was thoroughly tested by Rings and Lehnertz (2016) for networks of weakly chaotic Rössler oscillators, also in the presence of noise. The main result is that, for networks with high mean degrees and a larger number of nodes (*N* ≫ 10), the triplet analysis does not appear to provide information about directional couplings other than the one obtained with the pairwise analysis.

#### 2.3.4 Reconstruction of large networks

The generalization of the presented techniques to the case of large networks is straightforward. So, Pikovsky (2018) has treated three basic models of coupling in large networks (below, we assume that the initial preprocessing, including protophase extraction and a transformation to the phase, is completed):

$${\dot{\varphi }}_{k}=\omega_{k}+\overset{N}{\underset{j=1;j\neq k}{\sum }}\Gamma_{kj}\left(\varphi_{j}-\varphi_{k}\right),$$ (19)

$${\dot{\varphi }}_{k}=\omega_{k}+\overset{N}{\underset{j=1;j\neq k}{\sum }}Q_{kj}\left(\varphi_{j},\varphi_{k}\right),$$ (20)

$${\dot{\varphi }}_{k}=\omega_{k}+\overset{N}{\underset{j=1;j\neq k}{\sum }}\;\overset{N}{\underset{l=1,l\neq k,l>j}{\sum }}G_{\mathrm{kjl}}\left(\varphi_{j},\varphi_{k},\varphi_{l}\right).$$ (21)

Here Eq. 19 describes a so-called Kuramoto-Daido network with arbitrary and generally different pairwise coupling functions depending on the phase differences; Eq. 20 describes a network where pairwise coupling functions are general 2*π*-periodic functions of the phases; Eq. 21 describes a hypernetwork with triplet interactions.

As the first step, we calculate from the time series of phases their time derivatives ${\dot{\varphi }}_{k}$ [the most common approach here is to use Savitzky-Golay filtering, see Ahnert and Abel (2007)]. In the next step, we represent the coupling functions as sums of elementary sin and cos functions (in the case of the Kuramoto-Daido coupling) or as sums of products of these functions. For example, the coupling function *Q*_*kj*_ in Eq. 20 is represented as

$$Q_{kj}\left(\varphi_{j},\varphi_{k}\right)=\overset{2M}{\underset{m=1}{\sum }}\overset{2M+1}{\underset{l=1}{\sum }}q_{\mathrm{kjml}}f_{m}\left(\varphi_{j}\right)f_{l}\left(\varphi_{k}\right),$$ (22)

where

$$f_{2n-1}\left(x\right)=\cos nx,\;f_{2n}\left(x\right)=\sin nx,\;f_{2M+1}=1,\;n=1,\ldots ,M.$$

**FIGURE 3:**
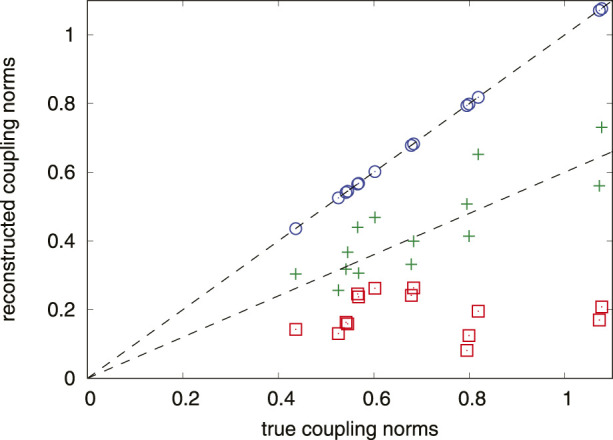
Results of the reconstruction of the coupling constants for one oscillator in the random network (Eq. 20) using 5,000 observation points. Norms of the reconstructed coupling functions are plotted vs. the true ones. Square, plus, and circle symbols correspond to *M* = 1, *M* = 2, and *M* = *p* = 3, respectively. Dashed lines help to see validity of a linear relation between the reconstructed and true norms. For the proper number *M* = 3 of explored harmonics the reconstruction is very good.

Parameter *M* defines the number of harmonics in the test functions; it should be adjusted according to complexity of the coupling function. Exploiting this representation, one performs minimization of the squared error for the discrete time series *φ*_*k*_(*n*)

$$\underset{n}{\sum }{\left({\dot{\varphi }}_{k}-{\tilde{\omega }}_{k}-\underset{j}{\sum }Q_{kj}\left(\varphi_{j},\varphi_{k}\right)\right)}^{2},$$

where *Q*_*kj*_(*φ*_*j*_, *φ*_*k*_) is substituted using Eq. 22, and then finds unknown coefficients *q* and frequencies ${\tilde{\omega }}_{k}$ by virtue of singular value decomposition. We illustrate the performance of the approach for the Winfree-type model with random coupling functions (containing *p* = 3 harmonics) and a fully connected random network of 16 oscillators in Figure 3.

Similar approaches to reconstruction of large oscillatory networks have been discussed by Casadiego and Timme (2018), Panaggio et al. (2019), Tokuda et al. (2019), Novaes et al. (2021), and Rings et al. (2022). We mention that there is still a problem of discriminating small couplings from nonexisting ones. Generally, one obtains false positive and false negative interactions. See Cecchini et al. (2021) for an extended analysis of relations of these errors to the network structure.

## 3 Case II: pulse-like oscillatory dynamics

Suppose we observe neuronal spiking, or the data we measure looks like spiking, so reducing the signals to point processes is appropriate. If it is reasonable to assume the self-sustained activity, then the proper model is that of pulse-coupled integrate-and-fire units^7^. In this one-dimensional model, the phase of each unit grows uniformly, ${\dot{\varphi }}_{k}=\omega_{k}$ . When the phase *φ*_*k*_ attains 2*π*, the unit issues a spike, and its phase is instantaneously reset to zero.

### 3.1 Undirected connectivity

Suppose we deal with two units and know the times at which they fire. Without loss of generality, we say that phase is zero when a unit spikes, and it grows to 2*π* within an inter-spike interval. The simplest way to quantify the degree of synchrony for two units is to approximate their phases linearly between the spikes and compute the synchronization index. For advanced techniques focused on analysis of spike train data, see Kreuz et al., 2007, Kreuz, 2011, and Satuvuori et al., 2017.

### 3.2 Directed connectivity

Here, we discuss the connectivity inference for the case when a network of pulse-coupled oscillators can reasonably model the system, and the available data represent sequences of spikes (Cestnik and Rosenblum, 2017). We assume that the coupling is sufficiently weak to justify the phase description. Next, we suppose that a unit’s phase response curve (PRC) is the same for all incoming connections, though PRCs of different units generally differ. We describe individual units by the integrate-and-fire model. It is, without interaction, the phase of each unit grows uniformly, ${\dot{\varphi }}_{k}=\omega_{k}$ . When the phase *φ*_*k*_ attains 2*π*, the unit issues a spike, and its phase is instantaneously reset to zero. At this moment, the unit *k* sends a stimulus through all outgoing links and thus kicks all units connected to it through these links. When unit *j* receives a kick from unit *k*, its phase is instantaneously reset according to its PRC *Z*_*j*_(*φ*):

$$\varphi_{j}\to \varphi_{j}+\varepsilon_{jk}Z_{j}\left(\varphi_{j}\right),$$

where coefficient *ɛ*_*jk*_ quantifies the strength of the link *k* → *j*. Generally, *ɛ*_*jk*_ ≠ *ɛ*_*kj*_.

In our approach, we choose one oscillator (let it be the first one) and consider all its incoming connections. To simplify the notations, we omit the index 1, so for the incoming links we rename *ɛ*_1*k*_ by *ɛ*_*k*_, with *k* = 2, 3, … , *N*. Using an iterative procedure described below, we infer this oscillator’s frequency *ω*, PRC *Z*, and *ɛ*_*k*_. Next, we perform the same analysis for all other units.

We denote the inter-spike intervals for the chosen (first) unit as *T*_*m*_, where *m* = 1, 2, … , *M* and *M* + 1 is the number of spikes in the pulse train of the first unit. We write the following equations for the phase evolution within each interval *T*_*m*_:

$$\omega T_{m}+\overset{N}{\underset{k=2}{\sum }}\varepsilon_{k}\overset{n_{m}\left(k\right)}{\underset{l=1}{\sum }}Z\left(\varphi_{m}^{\left(k,l\right)}\right)=2\pi .$$ (23)

Here $\varphi_{m}^{\left(k,l\right)}$ is the phase of the first unit at the instant when it receives the *l*th spike from the unit *k*, within the inter-spike interval number *m*; *n*_*m*_(*k*) is the number of incoming stimuli from the unit *k* within interval *T*_*m*_. For sufficiently large *M*, these equations can be solved by iterations to provide unknown *ω*, *ɛ*_*k*_, *Z*, see Cestnik and Rosenblum (2017).

The key idea for solving Eq. 23 is as follows. Suppose for a moment that we know phases $\varphi_{m}^{\left(i,l\right)}$ and coupling coefficients *ɛ*_*k*_. Representing *Z*(*ϕ*) as a finite Fourier series, we obtain the overdetermined linear system for unknown Fourier coefficients (provided *M* > 2*N*_*F*_ + 1, where *N*_*F*_ is the order of the Fourier expansion). Suppose, *vice versa*, that we know phases and PRC; then we obtain a linear system to find coupling coefficients *ɛ*_*k*_. These observations suggest a process to solve the problem: we start with some initial estimates for $\varphi_{m}^{\left(i,l\right)},\varepsilon_{k}$ and obtain the first estimates for *Z*, *ω*. Next, we use the first estimates for *Z*, *ω* to obtain second estimates for $\varphi_{m}^{\left(i,l\right)},\varepsilon_{k}$ , etc. Since the coupling coefficients and PRC enter the phase dynamics equation as a product, we obtain these quantities up to a constant factor. The numerical tests show that the iterative procedure converges for the random initial distribution of *ɛ*_*k*_ or equal values *ɛ*_*k*_ = *ɛ*. The initial phase is taken as linearly growing within the inter-spike interval. The next iterations are piece-wise linear: the phase grows linearly between the incoming spikes and changes instantaneously when these spikes arrive. Suppose we compute the phase at the end of an inter-spike interval for some iteration; we denote this phase as *ψ*_*m*,*i*_, where *m*, *i* label the inter-spike intervals and the iteration, respectively. *ψ*_*m*,*i*_ is calculated according to the left-hand side of Eq. 23, using *ω*, *ɛ*_*k*_, *Z* from the previous iteration. Since the latter values are not exact, generally *ψ*_*m*,*i*_ ≠ 2*π*. The outcome of this fact is twofold. First, we rescale the phase by 2*π*/*ψ*_*k*,*i*_. Second, we use the standard deviation *σ*_*i*_ = std(*ψ*_*m*,*i*_ − 2*π*) as a measure of convergence: if *σ*_*i*_ decreases with iterations, then the results are reliable. Furthermore, one can reconstruct the same network many times, starting from different initial values of the coupling strength and checking the convergence. If different initial values yield close results, then the latter can be trusted.

The tests demonstrate that the technique is robust and capable of dealing with relatively short data (several hundreds of spikes suffice). If the coupling is not weak enough, the estimation of the network connectivity remains correct, but the obtained PRC becomes amplitude-dependent. The method works well unless the nodes synchronize. It may also fail in case of sparse networks where one can expect purely periodic nodes. Indeed, the iterative procedure requires some variability in the drive. However, noise in realistic networks enhances the reconstruction.

## 4 Discussion

In this mini-review, we presented one line of a broad field of research aiming at network inference from observations. The presented approach explicitly relies on the coupled oscillation theory. It is, therefore, suitable only for the case where all network nodes are self-sustained oscillators, either noisy periodic or weakly chaotic. An additional crucial requirement is that each node is observed and the observables are appropriate for phase estimation. It is not easy to verify these conditions in practice, which is a weak point of the presented approach. However, if the assumptions used are correct, the approach allows for a straightforward interpretation of the results and yields a reasonable inference of the structural connectivity. As important applications, we mention analysis of the interaction between cardiovascular and respiratory systems in humans (Kralemann et al., 2013a) and between cardiac, respiratory, and delta brain rhythms in anesthetized rats (Musizza et al., 2007), as well as use of coupling functions to reveal microvascular impairment in treated hypertension (Ticcinelli et al., 2017). A difficult problem is the nonstationarity of real-world data, see Stankovski et al. (2012) for an estimation of time-evolving coupling. Finally, we mention the assessment of the statistical significance of the inference. The typical approach implies comparing the obtained results with those for the surrogate data, see, e.g., Andrzejak et al. (2003). A discussion of the corresponding techniques may be the subject of a separate review; here, we only mention a simple procedure exploited by Kralemann et al. (2013a) for the bivariate case. To analyze the significance of the coupling function estimation, they compared this function with those obtained for cardiac and respiratory time series taken from different subjects using a similarity index, which quantifies the similarity of forms of two two-dimensional functions.

Another popular approach exploits information-theoretical measures, see, e.g., Hlaváčková-Schindler et al. (2007), Faes et al. (2011), Xiong et al. (2017), and Krakovská et al., 2018. Multiple techniques from this group do not assume that one deals with self-sustained systems and have, therefore, a broad field of applications, e.g., they can be applied to networks consisting of both active and passive units. However, the interpretation of results is complicated. The detected information flows can be interpreted as functional connectivity, which is generally directed, because the information-theoretical measures are asymmetric, contrary to cross-correlations. This functional connectivity generally differs from genuine physical connections (i.e., the inference procedure can yield links that are not structural according to our definition). Finally, we mention a hybrid approach where one evaluates the directionality of interaction by applying the information theory approach to phases (Paluš and Stefanovska, 2003; Vlachos et al., 2022). For a review of techniques originating from different approaches, see Lehnertz (2011), Rings and Lehnertz (2016), Siggiridou et al. (2019), Moraffah et al. (2021), Shojaie and Fox (2022), and Runge et al. (2023). Unfortunately, a detailed comparison of all existing techniques is missing because it requires much effort, publically available codes, and well-designed test models.

## Footnotes

1 Explicit dependence means that there exists at least one partial derivative *∂H*_*l*_/*∂x*_*jm*_ that does not vanish identically, where indices *l*, *m* label components of **H** and **x**_*j*_, respectively.

2 Knowledge of the protophase suffices if only the frequency of the signal is required, since $\left\langle \dot{\theta }\right\rangle =\left\langle \dot{\varphi }\right\rangle$ , where ⟨⋅⟩ denotes averaging over time.

3 See Gengel et al. (2021) for the algorithm providing coupled oscillator phases.

4 We mention that the phase coupling functions *Q*(1,2) generally contain high-order terms in the coupling strength, cf. Eq. 5.

5 This representation assumes that *Q* has zero mean, which is generally false. The coupling function can have a constant term that scales with the coupling strength. If several observations for different though unknown coupling values are available, it is possible to infer the correct value of the natural frequency, see Kralemann et al. (2007) and Kralemann et al. (2008). In the case of only one observation, assigning the constant term of the r.h.s. to *ω* seems to be the only option.

6 Kernel density estimation in three dimensions is computationally inefficient and difficult to interpret.

7 Integrate-and-fire system is the simplest representation of a relaxation oscillator.
